# Unpacking Transient Event Dynamics in Electrophysiological Power Spectra

**DOI:** 10.1007/s10548-019-00745-5

**Published:** 2019-11-21

**Authors:** Andrew J. Quinn, Freek van Ede, Matthew J. Brookes, Simone G. Heideman, Magdalena Nowak, Zelekha A. Seedat, Diego Vidaurre, Catharina Zich, Anna C. Nobre, Mark W. Woolrich

**Affiliations:** 1 Department of Psychiatry, Oxford Centre for Human Brain Activity, Wellcome Centre for Integrative Neuroimaging, University of Oxford, Oxford, UK; 2 Sir Peter Mansfield Imaging Centre, School of Physics and Astronomy, University of Nottingham, University Park, Nottingham, NG7 2RD UK; 3 Nuffield Department of Clinical Neurosciences, Wellcome Centre for Integrative Neuroimaging, Oxford Centre for Functional MRI of the Brain, University of Oxford, Oxford, UK; 4 Department of Experimental Psychology, University of Oxford, Oxford, UK

**Keywords:** Electrophysiology, Spectrum, Dynamics, Bursting, Hidden Markov model

## Abstract

Electrophysiological recordings of neuronal activity show spontaneous and task-dependent changes in their frequency-domain power spectra. These changes are conventionally interpreted as modulations in the amplitude of underlying oscillations. However, this overlooks the possibility of underlying transient spectral ‘bursts’ or events whose dynamics can map to changes in trial-average spectral power in numerous ways. Under this emerging perspective, a key challenge is to perform burst detection, i.e. to characterise single-trial transient spectral events, in a principled manner. Here, we describe how transient spectral events can be operationalised and estimated using Hidden Markov Models (HMMs). The HMM overcomes a number of the limitations of the standard amplitude-thresholding approach to burst detection; in that it is able to concurrently detect different types of bursts, each with distinct spectral content, without the need to predefine frequency bands of interest, and does so with less dependence on a priori threshold specification. We describe how the HMM can be used for burst detection and illustrate its benefits on simulated data. Finally, we apply this method to empirical data to detect multiple burst types in a task-MEG dataset, and illustrate how we can compute burst metrics, such as the task-evoked timecourse of burst duration.

## Introduction

A wide range of brain regions generate spectrally specific rhythmic signatures in electrophysiology recordings. Whilst some of these ‘rhythms’ reflect genuinely sustained oscillatory activity, it has been raised that others may be better described as spectrally specific transient events (Jones 2016; van Ede et al. 2018). In the latter case, the dynamics may be better characterised by measures of the duration, rate, or amplitude of transient events, as they occur at the level of single trials, rather than standard static or trial-averaged estimates of power.

The transient perspective suggests that the typical picture of a persistent oscillatory response to, for example, a stimulus or during working memory, may be an artefact of averaging temporally variable, short-lived events across many trials (Lundqvist et al. 2016; Stokes and Spaak 2016). In some cases, the temporal dynamics of these spectrally specific events, rather than overall amplitude or power, may be the crucial variable. This perspective is prominent in several lines of research into the 13–30 Hz beta band. Visual attention is associated with increased beta activity consisting of 300–1000 ms bursts propagating throughout the visual stream (Wróbel 2000). Similarly, in motor cortex, single-trial analyses of 13–30 Hz beta power show motor task responses of several seconds that may be built from bursts of < 150 ms in both cortical and sub-cortical regions (Feingold et al. 2015; Sherman et al. 2016). The rate and timing of these burst-events predict somatosensory and motor performance (Little et al. 2018; Shin et al. 2017) suggesting that single-trial variability in these burst-parameters is likely to be more than noise. Finally, adaptive deep-brain stimulation targeted at reducing the duration of beta bursts, rather than continuous suppression of beta power in the subthalamic nucleus of patients with Parkinson’s Disease, may improve the efficacy and efficiency of DBS (Tinkhauser et al. 2017a, b). These converging findings provide evidence that the precise temporal dynamics underlying power changes in beta activity are functionally relevant across a range of brain regions, species and task contexts.

The temporal perspective that emphasises transient spectral events thus has a recognised value, but the estimation of their temporal metrics comes with a range of technical challenges. Accurate analysis of transient spectral events, or burst detection, necessitates the detection of power changes against background noise at the single-trial level. This is typically performed by thresholding the amplitude distribution of a bandlimited signal. For instance in two recent examples, Shin et al. (2017) used a threshold of six times the median beta power derived by wavelets, and Tinkhauser et al. (2017a) used the 75th percentile of the amplitude derived from the Hilbert transform. These thresholds are set to identify periods when a signal’s amplitude is in the long tail of a positively skewed amplitude distribution. However, a noisy signal is likely to have a near-Gaussian amplitude distribution with few outliers, which makes this threshold selection difficult or even arbitrary. In addition, the temporal dynamics of a signal are constrained by the time–frequency trade off in linear filters or time–frequency transforms (Aru et al. 2015; de Cheveigné and Nelken 2019). In other words, a high temporal resolution is desirable when analysing dynamics, but this leads to smearing in frequency, which makes it difficult to disambiguate between (potentially) different dynamics in neighbouring frequency bands.

Here, we briefly outline the challenges associated with inferring bursting events in time-series and how the problem may be approached with a Hidden Markov Model (HMM) in a data-driven manner. The HMM has previously been applied to MEG data acquired during a range of simple motor (Vidaurre et al. 2016), motor learning (Zich et al. 2018) and cognitive (Quinn et al. 2018) tasks. We expand on these papers by exploring the theory behind how an HMM might represent a transient spectral event in the context of the recent literature on bursting oscillations. We consider two HMM based methods which operationalise bursting phenomena in different ways and with different assumptions. The time-delay embedded HMM is then applied to a simple motor task to explore how temporal dynamics related to bursting might underlie electrophysiological power spectra. Firstly, in the case of between subject differences in beta power across a whole recording session and secondly for a contrast between pre-movement and post-movement time windows highlighting the post-movement beta rebound.

## Materials and Methods

### Temporal Dynamics and Spectral Power

Standard Fourier-based approaches describe times series with a set of pure sinusoids. This means that each frequency has a single amplitude and phase lag which is assumed to be constant over time. Following this, changes in spectral power are typically interpreted as a change in the magnitude or power of the underlying oscillation. This interpretation is unambiguous when comparing stationary sinusoids but can be misleading when the signals contain richer temporal dynamics. For instance, some oscillations particularly in the beta and gamma bands, may contain intermittent, ‘bursting’ activity; therefore it may be more appropriate to consider these as transient pulses of spectrally specific activity rather than tonic oscillations (van Ede et al. 2018). Crucially, changes in the temporal parameters of these spectral events, such as lifetime or rate of occurrence, may appear as changes in power in standard analyses. In comparing two signals, this may result in a false interpretation that one signal contains tonic oscillations with larger magnitude when instead it has longer or more frequent spectrally specific bursting events. Moreover, distinct changes in the underlying parameters (more bursts, longer bursts, or bursts with higher amplitude) may yield equivalent changes in trial-average power. Targeting the changes in the underlying parameters could therefore enrich our understanding (and help distinguish between alternative models) of power changes in various experimental conditions and clinical disorder.

A range of methods, including the HMM approach being proposed here, have been developed to describe the temporal dynamics that might underlie spectral power changes. These approaches are expository in their nature and, on their own, cannot unambiguously resolve the deeper question of whether the underlying physiology is comprised of transient events or oscillations occurring within noise. However, they can provide insight into the rich temporal structure that is missed with static or trial-averaged approaches.

**Fig. 1:**
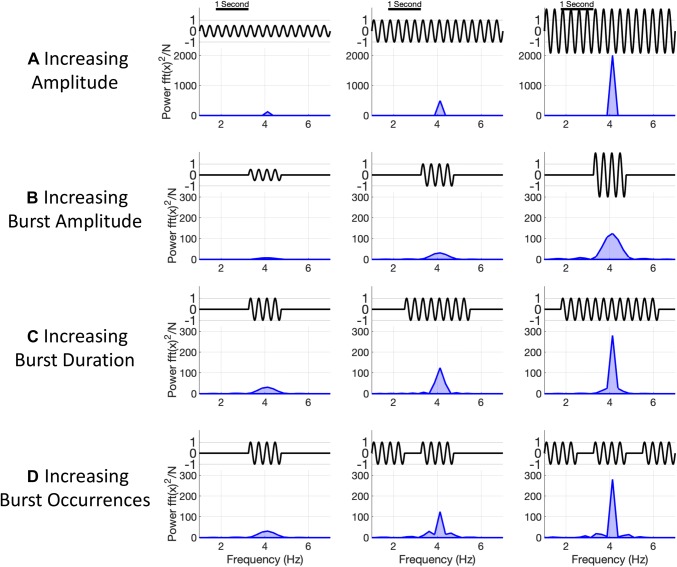
Illustration of how temporal dynamics can affect static power-estimates. **a** A tonic oscillation which increases in amplitude. In this instance, the peak in the power spectrum increases with each amplitude increase. Note that though the amplitude of the oscillations increases linearly, the peak in the power spectrum increases much faster. This is as power is related to the square of the amplitude of the oscillations. **b** A single burst which increases in amplitude. The increased amplitude leads to increases in the static power-spectrum even though only a single segment of the time-course is changing. **c** A single burst which increases in duration. The increased duration leads to large increases in the static power-spectrum which subjectively resemble the increases in **a**. However, in this case we might prefer to describe this change as increased duration rather than an increase in frequency domain power. **d** Finally, a burst of fixed duration which occurs more frequently. More occurrences have higher frequency domain power. As with **b**, we might prefer the temporal description that a burst occurs more frequently rather than overall power increases

Figure 1 illustrates changes in three features of a dynamic oscillation (i.e. amplitude, duration and occurrences) and how these changes affect power estimates (also see Fig. 3 in Shin et al. 2017). Figure 1a shows three short time-series containing tonic oscillations with increasing amplitudes. Correspondingly, the Fourier based power spectrum for each of these time-series shows successively higher magnitude peaks, leading us to correctly conclude that the amplitude of these cases is changing. Figure 1b shows a brief oscillatory burst, i.e. a single transient event (a short-lived period of time with non-zero amplitude), with increasing amplitude. The power spectrum computed across the whole window again indicates that power is increasing, though in this case only the power of a brief event is changing. More generally, the power or amplitude of an oscillation might be influenced by a range of factors. Figure 1c shows a dynamic signal containing a single transient event with a fixed amplitude but successively increasing duration. As with Fig. 1a, the power spectrum shows successively larger peaks around 4 Hz. Finally, the signals in Fig. 1d contain an increasing number of otherwise equivalent events, also resulting in increase of the total oscillatory power in the window. This brief simulation shows that increasing event amplitude, duration, or rate of occurrence all lead to increased magnitude peaks in the power spectrum. As such, if we only saw their static power spectra computed over the full time-window, we might conclude that all three cases show an increase in the amplitude of the underlying oscillation. We have the same issue when interpreting trial average changes in power which could arise from differing single-trial dynamics. Note that the sides of the spectral peaks for the four cases are slightly different. If our signal was a noise-free and sinusoidal, we could extract some extra information about dynamics from the side-bands of the main spectral peak. However, in a noisy, dynamics or short signal, it is extremely challenging to separate whether oscillatory signals, temporal dynamics or a combination of both are contributing to frequency domain power.

The increases in power seen in Fig. 1b–d are not mathematically inaccurate, they are a complete representation of the data under the assumptions of the Fourier transform. However, our interpretation of the power change in this form is ambiguous; we cannot make a strong statement about the which feature lead to our power change from the (non-time-resolved or trial-averaged) power spectrum alone. We need access to the temporal dynamics to resolve the difference between our three cases.

### Dynamics in Spectrally-Specific Events with Hidden Markov Models

One way to explore the temporal dynamics of transient events with specific spectral signatures, i.e. to do burst detection, is to use Hidden Markov Models. Our intention here is to provide an intuition into how the Hidden Markov Model can be used to operationalise and explore the temporal dynamics of transient spectral events, or bursts, rather than to make a formal comparison between all available alternative methodological approaches.

The Hidden Markov Model represents data as a system moving through a set of discrete states. Each state is an abstract representation linked to the data through a probabilistic observation model (Baker et al. 2014; Vidaurre et al. 2016). The observation model can take different forms to suit the data modality and features of interest (Vidaurre et al. 2018a). Previous applications of the HMM have used autoregressive models (Vidaurre et al. 2016) or multivariate Gaussian distributions (Baker et al. 2014; Quinn et al. 2018; Vidaurre et al. 2018a) as observation models describing multi-region or single-region data in human and animal data. This flexibility makes the HMM a practical choice for analysing a wide range of data types, by tuning the observation model whilst the essential mathematical framework and parameter inference remain the same.

In this paper, we will consider two HMM approaches to operationalise the detection of bursts of spectrally specific activity in single-channel (i.e. single-region electrophysiological data). In these two approaches, we either conceptualise a burst as a period of high amplitude, or as a period with a distinct power spectra.

#### Periods of High Amplitude

In the first approach, we define a burst as a period of high instantaneous amplitude within a predefined frequency band. These can be identified using an HMM with a Gaussian observation model inferred on amplitude envelope time-courses of bandpass filtered data. We refer to this as the amplitude-envelope HMM (AE-HMM; Baker et al. 2014; Quinn et al. 2018). In this approach, we typically specify that there are two states, one corresponding to the ‘burst’ state (i.e. when there is high amplitude), and one corresponding to a low amplitude state. A specific visit to the burst state then equates to the occurrence of a burst event.

This HMM variant is the most similar to power thresholding methods, in which a ‘burst’ occurs when the envelope exceeds a critical amplitude threshold. However, there are two key differences Firstly, the HMM does not threshold the amplitude envelope directly. Instead, each state has a probability of being ‘on’ at each moment in time. Switching between states can then be computed analytically by the Viterbi algorithm, or through a manual thresholding of the probabilities. The distributions of posterior probabilities from a well-fitted HMM tend to be strongly bimodal with most samples having probabilities close to zero or one. When selecting a threshold for the posterior probabilities by hand, this means that a relatively wide range of thresholds (typically between .33 and .66) tend to give similar results.

Secondly, the HMM applies temporal regularisation to avoid overfitting to small changes in the envelope. That is, the effect of having a transition probability matrix discourages too many spurious state transitions. This is particularly relevant when small changes in the envelope close to the threshold could lead to many small bursts being identified by a strict threshold. In contrast, brief dips during periods of otherwise high amplitude are less likely to lead to state changes in the HMM.

#### Periods with Distinct Power Spectra

We may also define a burst as a period of time with a distinct pattern of spectral properties (e.g. power). These can be identified using a time-delay embedded HMM (TDE-HMM; Quinn et al. 2018; Vidaurre et al. 2018b), which uses an HMM with a multivariate Gaussian observation inferred on a time-delay embedding of the wide-band data (i.e. a cross-temporal Gaussian distribution over some prespecified window). Each state then captures periods of time that have distinct auto-covariance. Though defined in the time-domain, the auto-covariance of a time series is closely related to its frequency content; the Power Spectral Density of a time-series can be computed from the discrete Fourier transform or its autocovariance (as shown by the Wiener–Khinchin theorem).

In this approach, we typically specify more than two states in the HMM. Where each state represents a particular type of spectral event, or bursting. For example, one state may detect bursts in the beta band, another in the alpha band. A specific visit to a state then equates to the occurrence of a burst event of the type represented by that state.

In contrast to the AE-HMM, the TDE-HMM states are defined by the magnitude and shape of an entire spectrum rather than the magnitude within a specified frequency band. Bursts can then be operationalised as short-lived states containing clear spectral features. As the TDE-HMM works on the whole spectrum from raw time-courses, there is no need for the tight bandpass filtering needed prior to computing the Hilbert transform in envelope-based methods. These filters require a priori specification and can themselves affect the dynamics visible in the data (de Cheveigné and Nelken 2019). As with the AE-HMM, the TDE-HMM is able to infer bursts without defining a priori thresholds.

#### HMM Estimation

The general theory of Hidden Markov Modelling is explored in Rabiner and Juang (1986) and Bishop (2006). The code to run the following simulations and real data analyses can be found on https://github.com/OHBA-analysis/Quinn2019_BurstHMM. These analyses were carried out in Matlab 2019a using the Signal Processing and Wavelet toolboxes.

HMM analyses are carried out using the HMM-MAR toolbox (https://github.com/OHBA-analysis/HMM-MAR; Vidaurre et al. 2018a, 2016). Previous HMM applications have detected transient events in MEG (Baker et al. 2014; Quinn et al. 2018; Vidaurre et al. 2018b, 2016), fMRI (Vidaurre et al. 2017) and simultaneous EEG-fMRI (Hunyadi et al. 2019).

### Simulated Examples

The AE- and TDE-HMMs are illustrated with a simulation. A noise time-course with a 1/f like profile is generated using direct-pole placement to define an order-1 AR model. Spectrally specific bursting events at either 20 or 35 Hz are added to this noise time-course at random intervals.

High amplitude burst events are detected using either a standard thresholding approach or a Gaussian HMM on the amplitude envelope estimated with the Hilbert transform after a narrow (15–20 Hz) or a wide (15–35 Hz) bandpass filter. For an informal comparison, a standard threshold is selected as twice the median amplitude envelope value in each example. The Gaussian HMM is specified to have two-states with the intention that the state with the higher mean amplitude reflects the bursting periods.

The same simulated time-course is used to illustrate the use of the TDE-HMM. In contrast to the AE-HMM, the TDE-HMM works on the raw time-courses, adaptively learning the spectral content, and therefore does not require a priori specification of the frequency bands of interest. A time delay embedding with lags from − 7:7 (58 ms) is defined, which means that each state has a [15 × 15] auto-covariance matrix as its observation model (the HMM state observation model means are set to zero). We fit the HMM with three states to reflect the periods of noise, low frequency bursts and high frequency bursts. State specific power spectra are computed for the TDE-HMM using a post hoc multi-taper spectral analysis (Vidaurre et al. 2016). The multitaper method computes the power spectrum multiplying the raw data with a set of seven orthogonal two-second data tapers (discrete prolate spheroidal sequences with a time-bandwidth product of 4) and taking the average spectrum over all the tapers (www.chronux.org; Mitra and Bokil 2007). The HMM-based multitaper approach used extends this method by weighing the contribution of each data point to the final spectral estimation using the state probabilities that the HMM inference provides for each time point in the dataset.

### Empirical Source-Space MEG Data

The TDE-HMM is applied to MEG sensor-space data acquired from 33 participants whilst completing a Go-NoGo task [full experimental details can be found in (Nowak et al. 2017)]. During each trial, participants prepare to make an abduction movement of the right index finger. On 80% of trials (Go trials) this movement is completed as expects and on the remaining 20% (NoGo trials) the prepared movement is no performed. Here we analysed the Go trials containing a valid finger movement. The sensor data were preprocessed with Signal-Space Separation Maxfilter before being converted to SPM12 format for processing in the OSL toolbox in MatLab. Each session is bandpass filtered between 1 Hz and 48 Hz and downsampled to 500 Hz. Independent Component Analysis (ICA) is used to identify and reject components of the sensor data relating to ECG and EOG. A single time-course is taken from the first principal component of 12 sensors over the left motor cortex as defined in (Nowak et al. 2017). This time course is epoched to the offset of the finger movement (identified from concurrent EMG recordings) to focus the analysis on the beta rebound. Finally, outlier trials are rejected from further analysis using an automated generalized extreme studentized deviate (GESD) algorithm.

Prior to HMM inference, the epoched data are downsampled by a factor of six to 83.3 Hz. This is as the TDE spectra will span the whole range from zero to Nyquist frequency. Therefore, downsampling will ensure that this range is focused on the frequency range of the physiological oscillations of interest. The TDE-HMM is inferred on the epoched data with six states and an embedding window of 23 samples (276 ms). We use a longer window here than for the simulations to allow the model to capture a greater range of auto-covariance structures in the data.

Once the HMM state time-courses and observation model have been inferred, we compute a range of temporal statistics from the state time-courses and extract the auto-covariance matrices from the observation models. The state-specific power spectra are computed directly from the observation model by taking the fast-Fourier transform (fft) of the middle row of the inferred autocovariance matrix. Next, the state time-courses are averaged across trials to compute a task-evoked fractional occupancy. HMM-regularised time–frequency transforms are computed from the outer product of the task-evoked fractional occupancy and the state-wise power spectra. This provides an alternative time–frequency transform of the data as seen by the inferred HMM. Finally, other task dynamics describing the transient spectral events, or bursts, can be computed. For example, we computed the task-evoked change in lifetimes for a state by creating a vector containing ‘NaNs’ (i.e. Not-a-Numbers) when the state is off, and the lifetime of the state visit when the lifetime is on. The average of these lifetimes across trials then summarises how the duration of state visits change over a trial.

## Results

### Amplitude-Envelope HMM (AE-HMM) Simulation

**Fig. 2:**
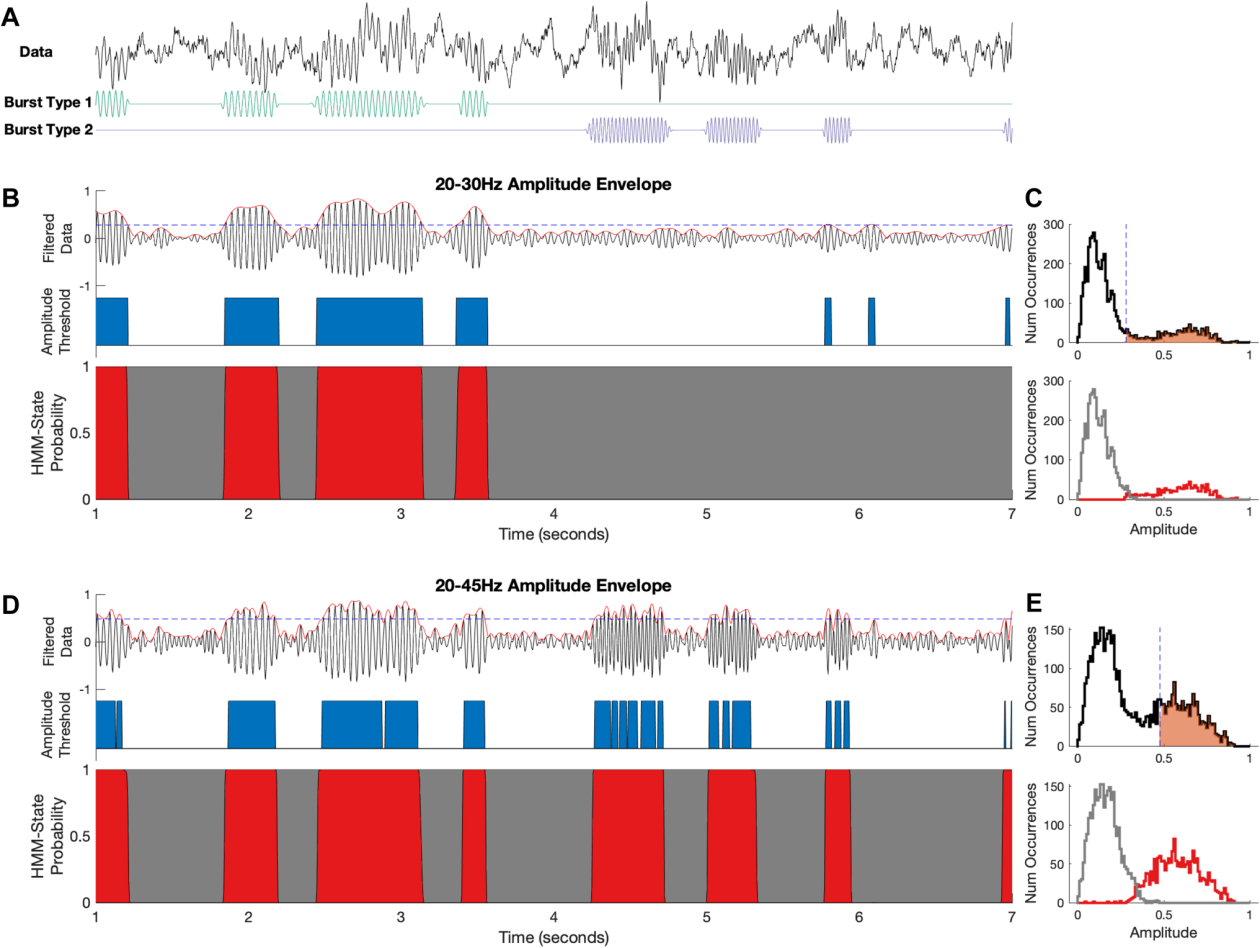
Using the amplitude-envelope variant of the Hidden Markov Model (AE-HMM) to detect transient spectral events that have high oscillatory amplitude in a predefined frequency band. **a** A simulated time-course containing 1/f type noise alongside two types of bursting: bursts of power at 20 Hz or 35 Hz. **b** The AE-HMM applied to a narrow-band amplitude envelope centred around 20 Hz. Burst events are estimated using amplitude threshold (blue) and AE-HMM-state time-courses (burst state in red). **c** The amplitude distribution of the time-course (black) with the simple threshold (dashed vertical line) and split by HMM states (grey and red). The temporal-regularisation of the HMM is apparent through the inference of overlapping amplitude distributions. **d** The AE-HMM applied to a wider band amplitude envelope containing both the 20 Hz and 35 Hz bursts. Burst events are detected by both the amplitude threshold and the HMM states. Both methods identify events around the correct times, though the amplitude threshold approach is noisier. Neither method can distinguish between the two types of bursts. **e** The amplitude distribution for the wider band signal, layout as in **c**

The simulated signal is shown in Fig. 2a; periods of oscillatory activity (i.e. Burst 1 and Burst 2) can be seen against the background noise. The AE-HMM is inferred with two states using Gaussian observation models, i.e. each state has its own inferred Gaussian distribution to model the distribution (across time points) of the amplitude envelope of the band-pass filtered signal. The state with the highest expected amplitude is considered to be the bursting state and highlighted in red in Fig. 2b, d.

First, results are shown when a narrow-band filter is applied prior to computing the amplitude envelope, such that the 35 Hz bursts are excluded. Figure 2b shows how both the HMM and the amplitude threshold are correctly able to identify periods of oscillatory activity for the remaining 20 Hz bursts, though the HMM’s performance does not critically depend on the pre-specification of a threshold value. The state-wise and thresholded amplitude distributions for the two approaches are shown in 2c. Whilst the amplitude threshold splits this distribution at a critical value (vertical dashed line), the two HMM state distributions (i.e. burst state in red, non-burst state in grey) are slightly overlapping reflecting the effects of the temporal regularisation in the HMM. This suggests that a brief period of lower amplitude during a high amplitude burst may not be enough for the AE-HMM to change state.

Next, results are shown when a wider-band filter is applied prior to computing the amplitude envelope, such that the both burst types are included. Figure 2d shows how both the HMM and amplitude threshold are able to identify general bursting around the correct time-periods, though the simple threshold provides a noisier estimate than the HMM in this wider band case. In Fig. 2d more high-frequency dynamics are present in the filtered envelope as this filter has a wider pass-band. This leads to the simple thresholding approach detecting many short bursts, or burst-splits, when the amplitude envelope dips below threshold. In contrast, the HMM is able to identify these as continuous periods of high spectral amplitude. This is further illustrated in Fig. 2e, in which the HMM state amplitude distributions are overlapping between the low and high-power states. This reflects the effects of the temporal regularisation within the HMM. When the envelope is close to the threshold, the HMM will not change state for a tiny fluctuation above or below it. This prevents a long but small amplitude event being split into several shorter events, which can occur when using standard thresholding methods.

While the AE-HMM approach does not require definition of the threshold a priori, neither AE-HMM nor amplitude thresholding is able to distinguish between the two different types of bursts, with their different carrier frequencies. This requirement of correctly specifying the frequency band(s) of interest a priori when using the AE-HMM is a limitation shared with standard thresholding methods.

### Time Delay Embedded HMM (TDE-HMM) Simulation

**Fig. 3:**
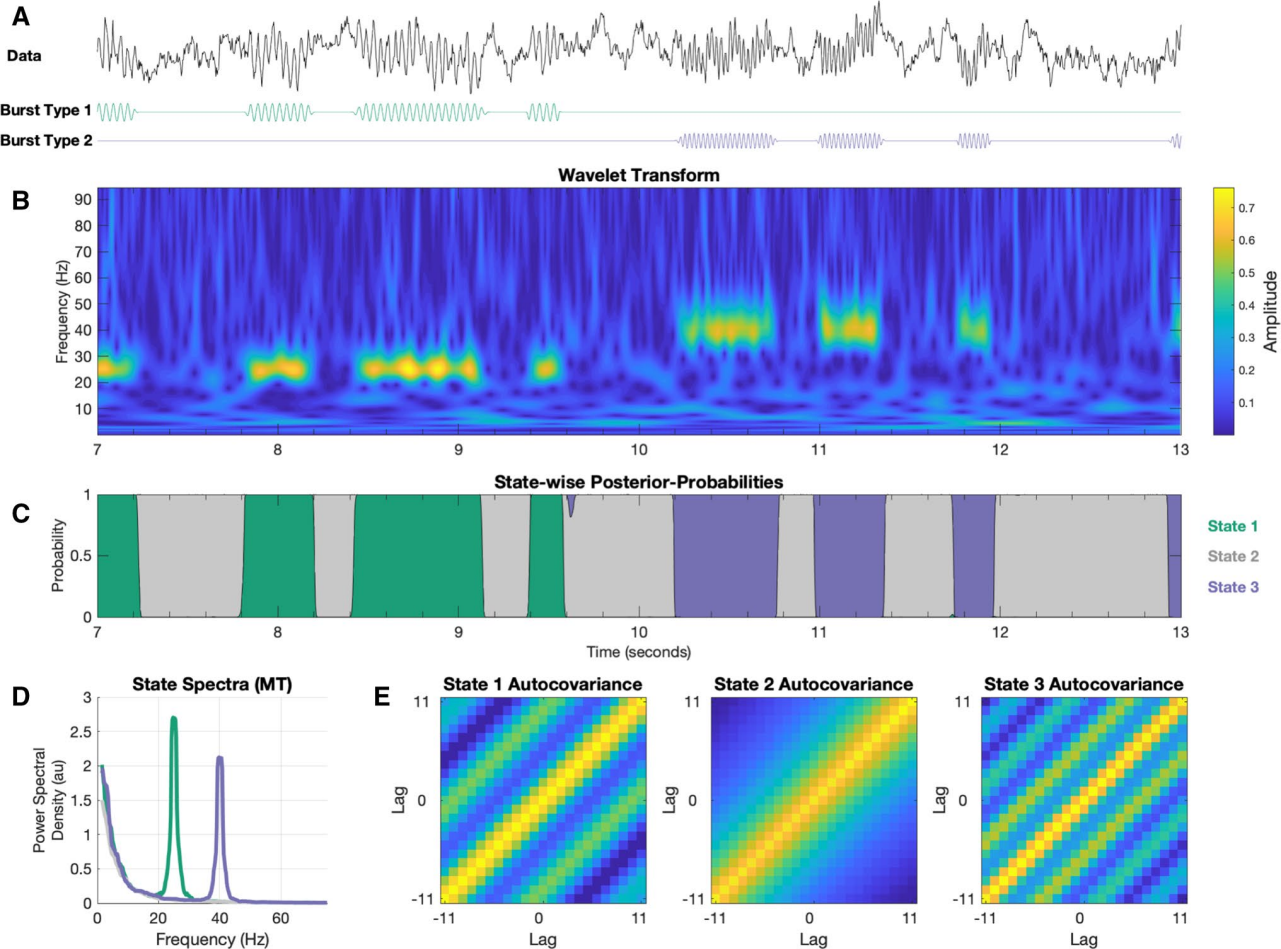
Using the time-delay embedded Hidden Markov Model (TDE-HMM) to detect transient spectral events. **a** A segment of a simulated time-course containing 1/f type noise alongside two types of bursting: bursts of power at 20 Hz or 35 Hz. **b** The wavelet transform of the simulated time-course showing the bursts occurring at different frequency bands. **c** The HMM state time-course from the inferred three states. States 2 and 3 correctly identify the periods of bursting activity. Crucially, the two types of bursts with their different frequency content are isolated into separate states. **d** The state-specific power spectra. States 2 and 3 have clear peaks reflecting the bursts associated with each state. State 1 reflects 1/f type noise. **e** The state-wise auto-covariance from the inferred HMM. The frequency content of each state (shown in **d**) is visible in the strong diagonal bands in the auto-covariances

Figure 3a, b show a segment of the simulated signal and its wavelet transform respectively. The transient periods of spectrally specific activity are visible in the wavelet transform, but their amplitudes and carrier frequencies show subtle changes over time due to interaction with the noise time-course. The HMM state time-courses for the three inferred states are shown in Fig. 3c. Here, states 1 and 2 identify the periods of low and high frequency activity and accurately reproduce the occurrence of the corresponding ground-truth bursts of the two different types. As the TDE-HMM has access to the whole frequency spectrum, unlike the AE-HMM and the simple thresholding approach, it is able to disambiguate between burst events of different frequencies within a single model. This can be seen in the state-wise power spectra in Fig. 3d, which show clear peaks at 20 and 35 Hz in states 1 and 2 respectively. State 3 reflects the 1/f background noise. Finally, the auto-covariances in the observation models show how the TDE identifies spectrally-specific activity. The diagonal bands in states 2 and 3 correspond to the structure of the autocorrelation of 20 and 35 Hz activity respectively. State 1 shows a relatively monotonically decreasing auto-correlation function with no sign of an oscillatory peak, consistent with the 1/f background noise.

The wider band state-specific spectra inferred with the time-delay embedded HMM allows us to extend the definition of a burst to a period with a distinct power spectrum rather than a period of high amplitude. This enables us to distinguish between events with different frequency profiles within the same model.

### Application to Empirical Source-Space MEG Data

#### TDE-HMM States Describe Time-Windows with Distinct Power Spectra

**Fig. 4:**
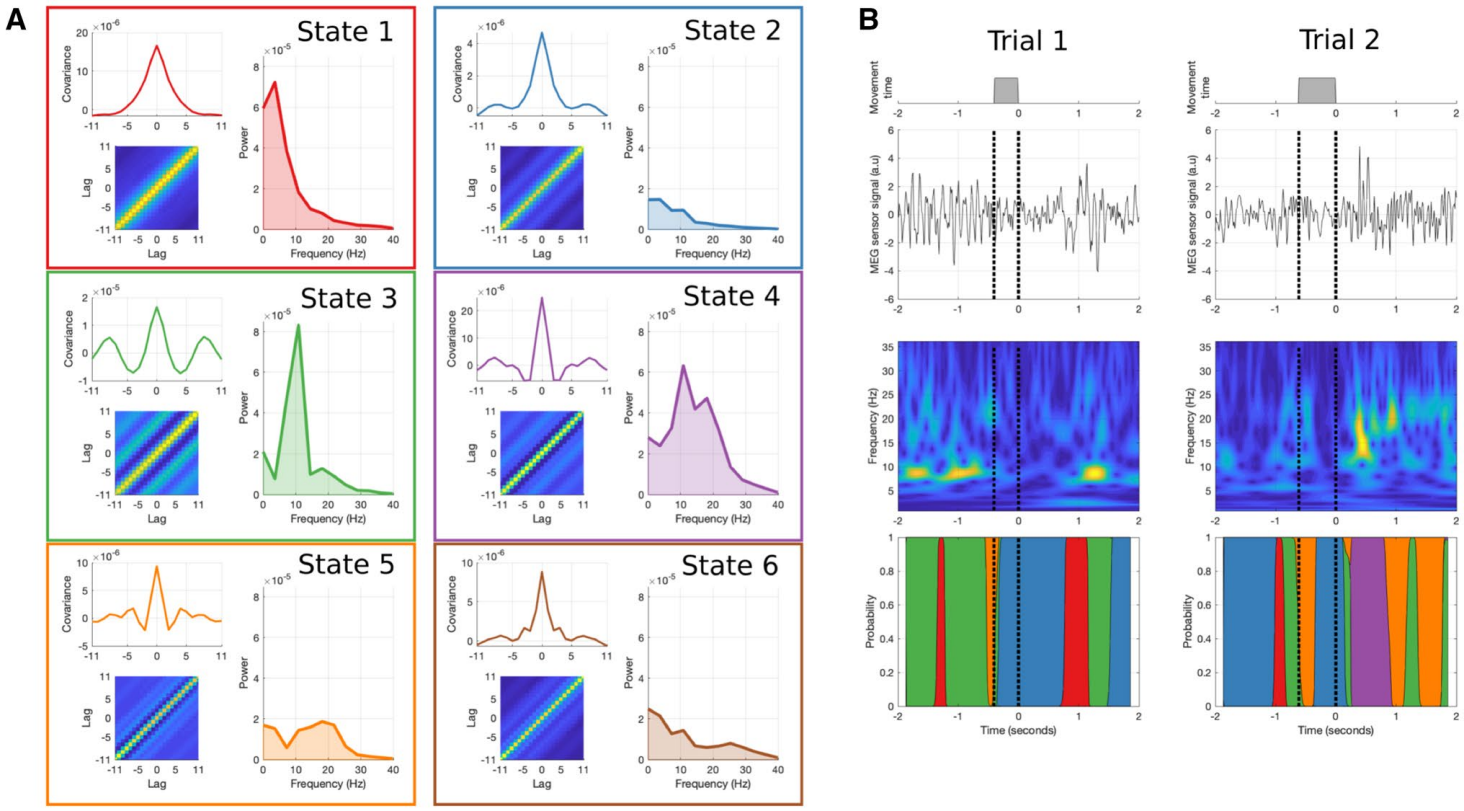
The application of the time-delay embedded Hidden Markov Model (TDE-HMM) to detect transient spectral events in real sensor space MEG data. Results are obtained by combining data across all trials (epoched to movement offset) and runs. **a** The inferred observation models for the six HMM states. Each box contains three sub-figures. Top-Left—the state autocovariance function as a function of embedding lag, taken from the centre row of the autocovariance matrix. Bottom-Left—the whole state autocovariance matrix. Right—the state power spectrum computed from the fft of the autocovariance function. **b** Two example Go trials. Each plot shows the movement duration (from onset to offset of abduction of right index finger), the trial time-course, time–frequency transform using wavelets and the HMM state posterior probabilities

The state observation models are summarised in Fig. 4a. These show the autocovariance function, autocovariance matrix and power spectrum for each state. The power spectrum is computed directly from the fft of the autocovariance function and show wide variability between states. The states with most diagonal bands in the auto-covariance matrices show relatively strong spectral peaks in the between 5 and 20 Hz. States 1 and 3 capture strong oscillatory peaks around 5 Hz and 10 Hz respectively whereas state 4 has a wide spectral peak covering both the alpha and beta bands (10–20 Hz) and state 5 has peaks in low frequencies (< 5 Hz) and the low beta bands (~ 20 Hz). Finally, states 2 and 6 have low variance 1/f type spectral shape. Figure 4b shows two trials alongside their wavelet transform and state time-courses. Several burst-like patterns of alpha- and beta-like activity can be seen in the wavelet transform. The states rapidly switch throughout the time-course and we can qualitatively see that each state is likely to correspond to different spectral features. Note the alpha bursts captured by state 3 in the first trial and the ~ 20 Hz beta activity captured by states 4 and 5 in the second trial. Both trials have windows of low variance without prominent oscillations indicated by state 2 being ‘on’.

#### HMM State Visits Show Rapid Dynamics Which Contribute to the Overall Power Spectrum

**Fig. 5:**
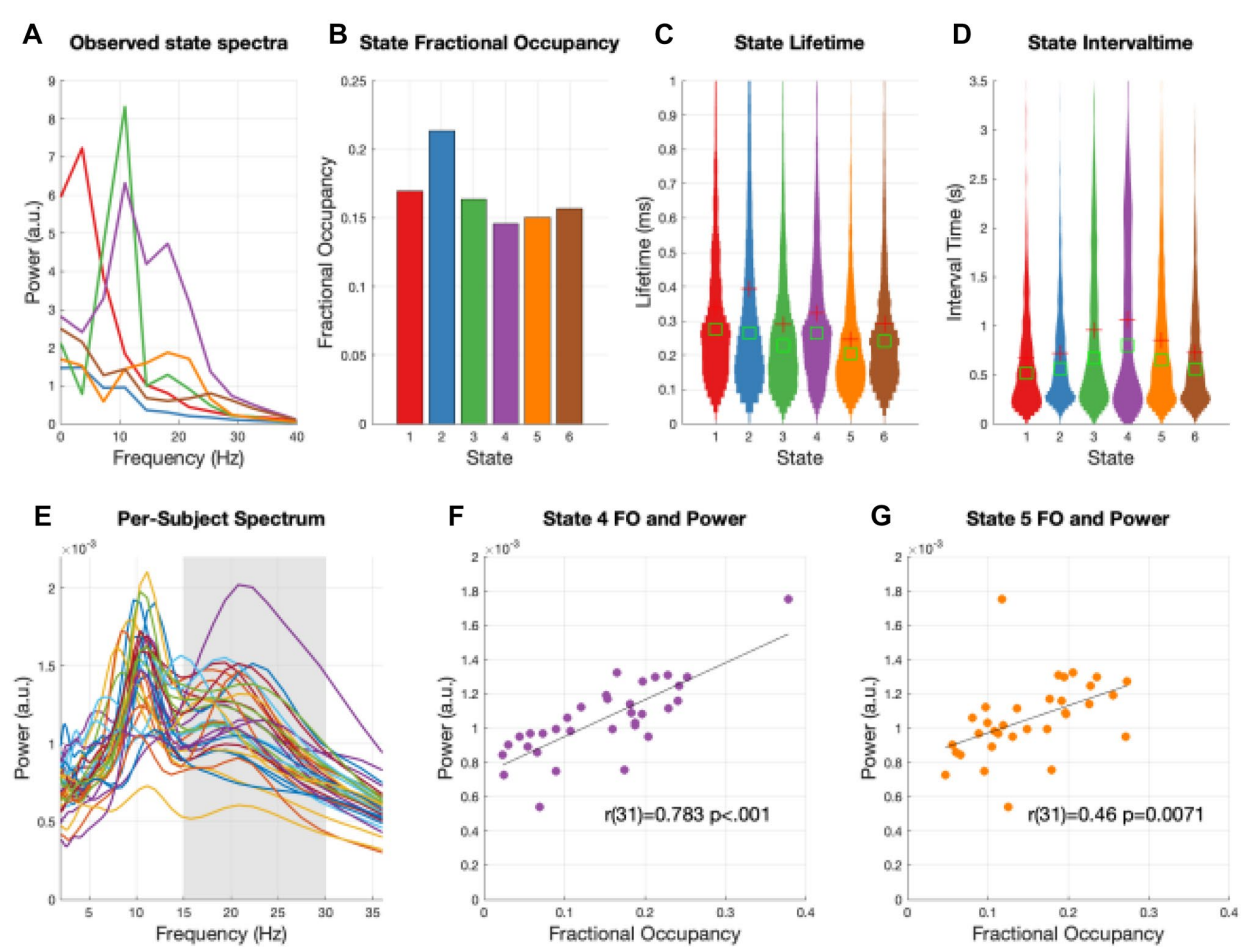
The non-task related temporal dynamics of HMM states. **a** The state power spectra computed from the fft of the autocovariance matrix. **b** The fractional occupancy of the six states. This is computed as the total duration of all visits to a specific state divided by the total duration of the time course. **c** The distribution of state lifetimes. **d** The distribution of state interval times, this is the duration of time between visits to a state. **e** The overall non-task related power spectrum for each participant. The grey box indicates a 15–30 Hz beta range. **f** The correlation of beta power (from **e**) with the fractional occupancy of state 4 across all participants. **g** The correlation of beta power (from **e**) with the fractional occupancy of state 5 across all participants

In the HMM framework, each state represents a distinct type of bursting with a specific power spectrum (Fig. 5a), and each state-visit represents an individual burst event. We can therefore use the inferred HMM state time-courses to estimate burst metrics associated with each state; such as the fractional occupancy, burst duration (state lifetime) and interval time (duration between state visits). Once computed, these can be contrasted between different time-windows, brain-regions or participant groups as a test of whether the temporal dynamics of spectrally-specific events might be changing. Taken alongside the standard power spectrum, these metrics allow us to disambiguate the different cases in Fig. 1. In our case, the fractional occupancy shows that each state is visited a roughly similar amount of time during the time-course (Fig. 5b). There are some differences between states, with the low variance 1/f type state 2 having the largest occupancy. All states have average lifetimes around 100–300 ms (Fig. 5c) and interval times (duration between state visits) of around 500–1000 ms (Fig. 5d). However, there is considerable variability in these metrics. Some state visits are as short at 50 ms, whilst a small number of visits last for closer to 1 s. Finally, These distributions are close to those seen in previous applications to resting-state and task data (Quinn et al. 2018; Vidaurre et al. 2016, 2017).

As suggested by the simulation in Fig. 1, these temporal statistics can directly relate to the standard power spectrum. Cross-subject variability in 15–30 Hz beta band power (Fig. 5e) is strongly correlated with cross-subject variability in the fractional occupancy of states 4 and 5 (Fig. 5f, g respectively). This suggests that participants with the strongest overall beta power also spend the longest time in the states with strong beta power.

#### Task Evoked Dynamics in HMM States

**Fig. 6:**
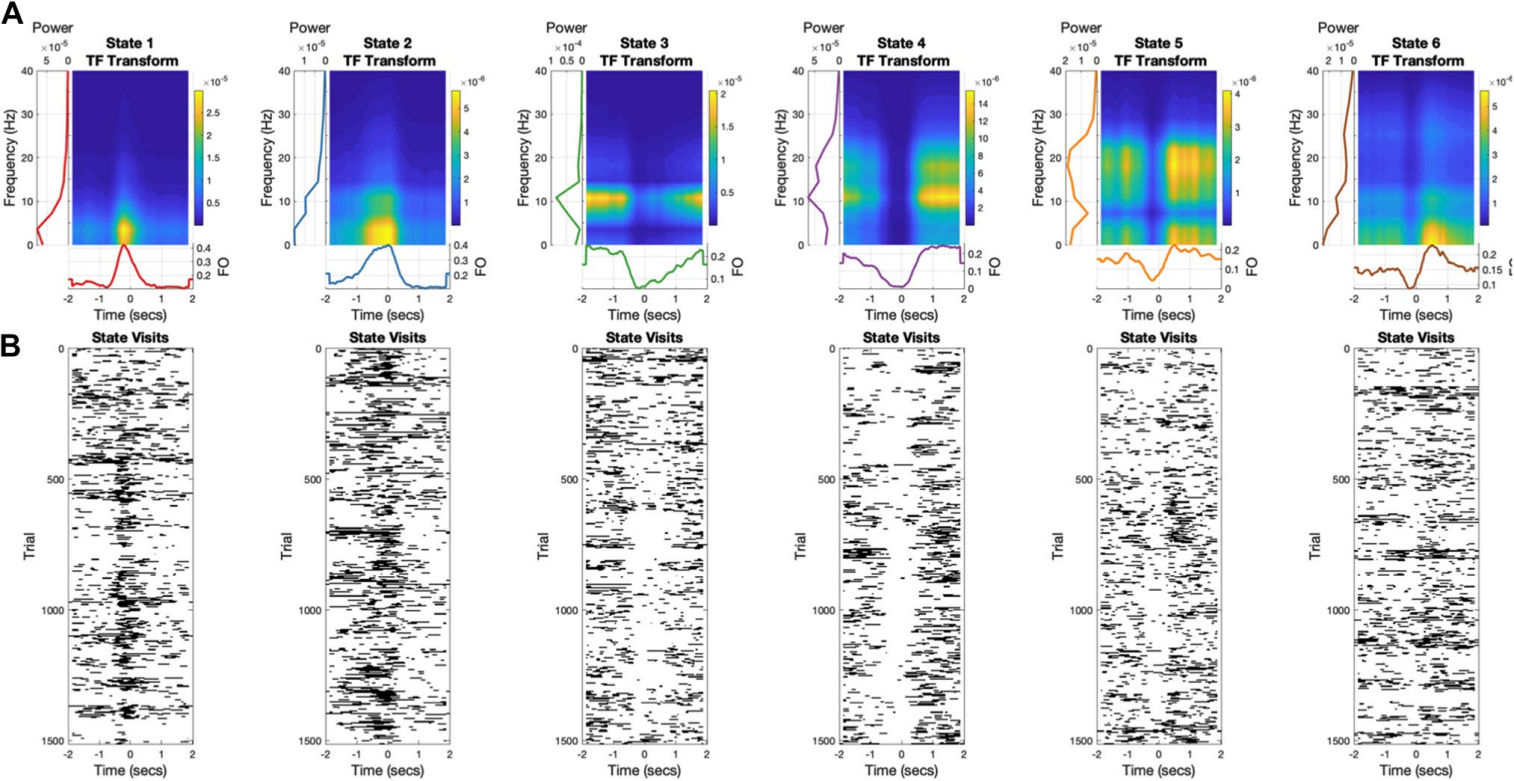
Task dynamics for each of the six inferred HMM states. Though the average time–frequency transform of each state presents a clear picture of its dynamics across each trial, the individual trials show wide variability in the number, duration and interval time of each state. Each subfigure **a**–**f** contains two sub-figures. Top—the HMM regularised time–frequency response for the single state. This is computed from the outer product of the frequency response (left) and the task-evoked fractional occupancy (bottom). Bottom—the state visits for all trials across all participants. Times when the state are ‘on’ are marked in black. The task-evoked fractional occupancy is computed from the average of this matrix across all trials

Though the HMM inference was blind to task timings, we can look at any task dependent changes in the state dynamics by averaging the state-time courses across trials as we would with the data during a typical evoked response (ERP/ERF) analysis (e.g. an evoked state response). An HMM-regularised time–frequency plot can then be constructed by the outer product of the evoked state-time course and the state-wise power spectrum (Vidaurre et al. 2016). Figure 6A shows these HMM-regularised time–frequency plot for each state. When averaged across trials, state shows clear time–frequency dynamics with a range of relatively transient (states 1 and 2) and more sustained responses (states 4 and 5). The raster-plots showing the occurrences of each state for all trials (Fig. 6b) reveal the trial-by-trial variability captured by the HMM. Though the broad patterns in the task evoked fractional occupancy time-courses are visible, all states show wide variability in the temporal onset and duration of state visits between trials.

**Fig. 7:**
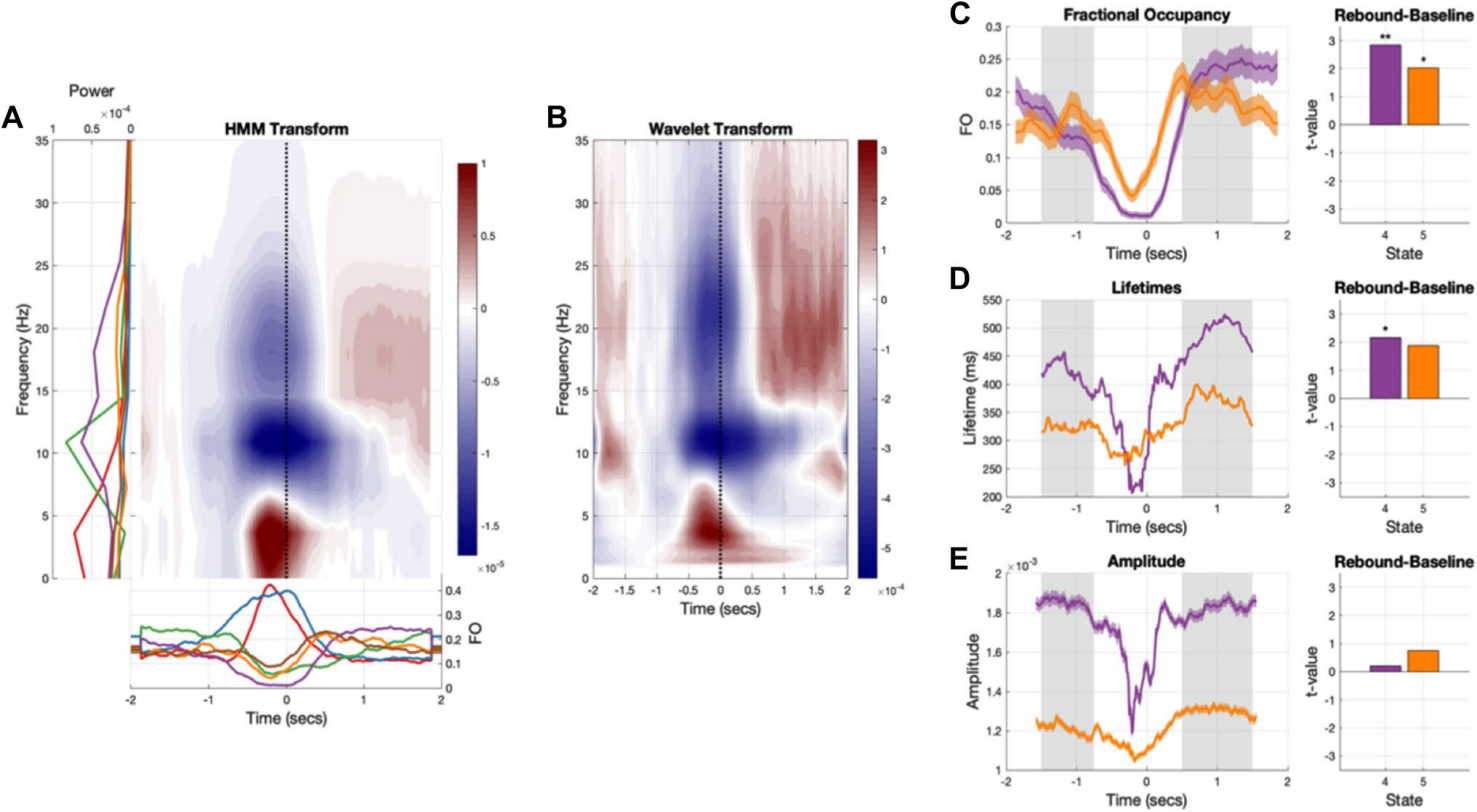
The task-related HMM dynamics. The HMM is able to reproduce the key features of a standard time–frequency decomposition and shows that the beta rebound effect is driven by increased occurrences of states 4 and 5, and increased lifetimes of state 4. **a** The HMM regularised time–frequency transform of the task data, computed from the outer product of the task-evoked fractional occupancies (bottom) and the state power spectra (left). **b** The baseline-corrected wavelet time–frequency transform of the task data. **c** Left—the fractional occupancy of states 4 and 5 across the trial. The shaded bars indicate the standard error of the mean across participants and the grey region indicates the baseline (− 1.5 to − .75 s) and beta rebound (.75 to 1.5) seconds. Right—t-statistics for a contrast between the baseline and rebound period for each state. Both states show a significant increase in occupancy during the rebound period relative to baseline (** indicates *p* < 0.01 and * indicates *p* < 0.05). **d** Left—the average lifetime of states 4 and 5 across the trial. The shaded bars indicate the standard error of the mean across participants and the grey region indicates the baseline (− 1.5 to − .75 s) and beta rebound (.75 to 1.5) seconds. Right—t-statistics for a contrast between the baseline and rebound period for each state. State 4 shows a significant increase in visit lifetime during the rebound compared to baseline. **e** Left—the average amplitude of states visits for states 4 and 5 across the trial. The shaded bars indicate the standard error of the mean across participants and the grey region indicates the baseline (− 1.5 to − .75 s) and beta rebound (.75 to 1.5) seconds. Right—t-statistics for a contrast between the baseline and rebound period for each state. Neither state shows a significant difference in visit amplitude compared to baseline

The HMM-regularised time–frequency spectra are baseline corrected (− 1500 to − 750 ms before movement offset) and summed across all states (Fig. 7a) to compare with a traditional wavelet time–frequency analysis (Fig. 7b). The HMM time–frequency transform is able to capture the dynamics shown by the wavelet transform. Both show a strong evoked response and 10–25 Hz decreased power after movement onset followed by a 15–25 Hz rebound after movement offset. Crucially, the HMM allows for further investigation into the single trial dynamics (number of occurrences, event lifetime, event amplitude), which underly the power spectral changes observed in the HMM-regularised time–frequency spectra and traditional wavelet time–frequency spectra.

#### Temporal Dynamics in HMM States Contribute to Movement Induced Power Changes

The trial-wise dynamics of the temporal statistics estimated from the HMM allow us to probe the potential scenarios in Fig. 1 in a task setting. Here, we focus on how two states with peaks in the 15–30 Hz beta band (see Fig. 4a) change between a baseline (− 1500 to 750 ms, relative to movement offset) and post-movement beta rebound (500–1500 ms, relative to movement offset) window. Figure 7a, b show a strong increase in beta power between these windows, though they do not tell us which dynamic features underly this change. Here, we explore the beta rebound, an increase in 15–30 Hz power after the offset of a movement typically described with trial-averaged wavelet transforms. Using the HMM states we look at whether this power change could arise from a change in the amplitude, duration or rate of occurrence of bursting events (see Fig. 1) described by the HMM.

The task evoked fractional occupancy is computed by averaging the state post-probabilities across trials (Fig. 7c). In this case, the evoked fractional occupancy tells us the proportion of trials in which each state was active at each given time point. Both states show a significant increase in fractional occupancy in the rebound window compared to baseline, suggesting that an increased number of state events is contributing to the increased power in the beta rebound. Both states show a sharp drop in occupancy during the movement time itself. We further investigate how state lifetime changes over the trial by taking the state time course (containing ones when the state is ‘on’ and nans when the state is ‘off’) and replacing the ones in each state visit with the lifetime of that visit. We can then average this across trials to get a task-evoked state lifetime, similar to the evoked fractional occupancy. State 4 shows a sharp decrease in lifetime during movement and a significant increase during the rebound. In contrast, state 5 has modest but not significant changes between the baseline and rebound windows. The trial average perspective shows that the beta rebound continues for 1.5 s after movement offset (limited by the duration of our trial) the HMM suggests that this is built from brief state visits of 200–500 ms. Finally, we can compute a state-amplitude time-course similar to the evoked lifetimes. Here we replace the ones in the state time course with the observed beta power from a wavelet transform during each visit. Neither state shows a significant change in amplitude between the baseline and rebound windows.

In sum, the HMM representation allows us to explore the temporal dynamics underlying change in power during a task response. In this case, we can show that increased 15-30 Hz power during the beta rebound arises from increased number of visits to states 4 and 5, and an increased lifetime of visits to state 4. This analysis, alongside previous findings, shows that HMM state dynamics can describe complex task dynamics with transient spectrally-specific events such as the ones in this paper (Quinn et al. 2018; Vidaurre et al. 2016). Critically, by targeting the single-trial events, they also allow an analysis of the single-trial parameters that constitute such dynamics, providing greater granularity at the individual trial level than conventional measures of time-resolved trial-average spectral power.

## Discussion

The Hidden Markov Model provides a powerful approach for operationalising the detection of transient spectral events, or bursts of activity, in neuronal time-courses. The time-delay embedded (TDE) HMM is able to concurrently detect different types of bursting across a wide frequency range without the need to predefine a frequency bands of interest, and in general the HMM is able to detect events without a priori threshold specification. The model provides a rich description of both spectral content and temporal dynamics, which can be interrogated to explore the single-trial constituents of trial-averaged neural activity and how they are modulated by different experimental and clinical conditions.

### Selecting Between HMM Variants

Several factors affect the choice of which HMM variant to use for a given analysis. The AE-HMM is sufficient for cases when interrogating amplitude dynamics within a known frequency band, but the spectrally-resolved approach of the TDE-HMM case is preferred where the precise spectral content of interest is not known and where there may be multiple burst types. The present applications are limited to detecting events in the 1–48 Hz frequency range as higher frequency gamma oscillations can be challenging to detect. Future work will extend the use of the TDE-HMM and HMM-MAR to detect transient events in the gamma range from broadband signals.

As an alternative, the autoregressive HMM can also be used to explore spectrally specific state dynamics (HMM-MAR; Vidaurre et al. 2016). This defines observed states with distinct autoregressive coefficients (AR). Like the auto-covariance, the AR coefficients are closely related to the power spectrum of the data and can be used to directly compute the Power Spectral Density (PSD). As the HMM-MAR states are defined by distinct autoregressive models, they will also have distinct power spectra. We would expect similar results from the HMM-AR approach. We have used the TDE as it typically performs more robustly, in particular with the shorter time-courses that we have in the MEG dataset used here.

### HMM Hyperparameter Selection

A few parameters need to be pre-specified prior to HMM inference. Firstly, a fixed number of states to infer must be defined. A larger number of states provides a finer-grained description of the data but may be more difficult for the software to infer and for us to interpret. This choice should be informed by the question at hand and the level of detail required by the analysis. For instance, two states are sufficient and provide the most straightforward approach for describing changes in narrow-band amplitude envelopes (as in the case of the AE-HMM), treating these like ‘on’ and ‘off’ states. However, allowing only two states would be limiting for the more complex full-spectrum states in the TDE-HMM (in our example we used six). The number of delay-embeddings in the auto-covariance to include in the TDE states must also be defined. Larger auto-covariance matrices allow for more time lags and therefore more structure in the data to be revealed.

The HMM can be sensitive to relatively subtle noise sources. Change in non-neuronal noise or variance in the time-course are likely to be modelled by one or more HMM states. Therefore, particular care should be taken during data pre-processing to ensure that the HMM states are characterising the interesting, neuronal variance in the signal (Quinn et al. 2018). Finally, as it happens with other models (e.g. ICA), the HMM inference has some stochasticity; therefore, the results can slightly vary between inference runs. It is advised to repeat the HMM inference several times to ensure that specific results are consistent.

### Statistical Assessment

Statistical testing of the HMM derived burst features can be carried out straightforwardly. Typically focusing on either group differences or task evoked changes in temporal dynamics. Group differences in temporal dynamics can be computed between different scan sessions or subjects within the dataset. Single subject features such as fractional occupancy or state lifetime can be computed from the inferred HMM and carried forward into group level statistics in the same way as standard between-subject variables. For example, this approach has been applied to identify group differences between patients with Alzheimer’s disease and healthy controls in a whole-brain AE-HMM (Sitnikova et al. 2018). Secondly, task or stimulus evoked changes can be computed by epoching the state time-courses and computing evoked state features. Group level GLM analysis of task evoked-fractional occupancies computed from MEG data during a face-perception task show rapid task responses which distinguish between face and non-face stimuli (Quinn et al. 2018).

### Summary and Conclusions

We have illustrated the use of an amplitude-envelope HMM to detect periods of high amplitude without pre-specification of an a priori amplitude threshold. Further, the time-delay embedded HMM was shown to be able to concurrently detect multiple types of bursting, each with distinct spectral profiles. Each of these methods estimate the temporal dynamics of spectrally specific events as a state time-course, where each state represents a distinct type of bursting, and each state-visit represents an individual burst event. This can then be used to estimate task response time-courses of burst metrics such as the rate of burst occurrence, burst duration, burst amplitude and interval time.

Whether this temporal dynamic perspective resembles underlying neuronal physiology more closely than sustained oscillation is the subject of ongoing research (van Ede et al. 2018). Nevertheless, there is utility in characterising the rapid temporal features of underlying frequency domain effects with models assuming transient state-visits rather than sustained effects (Quinn et al. 2018; Vidaurre et al. 2016). Novel burst-specific parameters such as rate of occurrence, duration and amplitude are being shown to have clinical (Tinkhauser et al. 2017a) and cognitive (Feingold et al. 2015; Shin et al. 2017) relevance. Finally, the spectrally-resolved HMM variants allow us to compute HMM-regularised time–frequency transforms which recreate reasonable time–frequency responses build from transient spectrally-specific events.

The HMM provides an effective technique for characterising such transient spectrally-specific events, bypassing some of the limitations of amplitude-thresholding approaches. Here, we have presented the relevant theory and motivations behind the use of the HMM as a potential ‘burst-detector’, and have presented an introduction to the available analysis scripts associated with this approach using the HMM-MAR toolbox.
